# A novel intelligent control of discrete-time nonlinear systems in the presence of output saturation

**DOI:** 10.1016/j.heliyon.2024.e38279

**Published:** 2024-09-21

**Authors:** Xuejun Zhou, Ying Dai, Ebrahim Ghaderpour, Ardashir Mohammadzadeh, Pierpaolo D'Urso

**Affiliations:** a College of Physics and Electronic Information, Yan'an University, Yan'an, 716000, Shaanxi, China; b School of Architecture and Civil Engineering, Shenyang University of Technology, Shenyang, 110870, Liaoning, China; c Department of Earth Sciences, Sapienza University of Rome, 00185, Rome, Italy; d Department of Electrical and Electronics Engineering, Sakarya University, 54050, Sakarya, Türkiye; e Department of Social Sciences and Economics, Sapienza University of Rome, 00185, Rome, Italy

**Keywords:** Type-3 fuzzy system, Discrete-time nonlinear systems, Output saturation, Convergence analysis

## Abstract

In this paper, a model free control method for a class of discrete time nonlinear systems is introduced. A type-3 fuzzy system estimates the unknown parameters required by the control system. The control system only uses the input and output data of the plant and therefore does not need to know its mathematical equations. On the other hand, the phenomenon of output saturation is a challenging problem for all control systems, addressed in detail in the proposed method. The convergence of the proposed method is guaranteed, and the control system is very robust in the face of changes in the dynamics of the plant. The simulation results on discrete-time nonlinear systems show that the proposed method is very accurate despite the high speed of convergence. In addition, the proposed method is robust for modeling uncertainties and has a better root mean square error and step response time compared to the other methods. Also, a comparison has been made between type-1 to type-3 fuzzy systems and control system based on trial and error, which shows firstly the importance of the presence of fuzzy system and secondly the superiority of type-3 fuzzy system compared to the other two types.

## 1 Introduction

Today, due to the advancement of technology, industrial systems have become very complex and interconnected because of these complexities, modeling these systems with linear low-order linear and hybrid models has become very difficult and impossible [[1], [2], [3]]. This inability to accurately model systems will reduce the efficiency of model-based control methods [4,5]. Also, in many of these systems, only limitations such as: saturation at the input, saturation at the output, external noise, system uncertainty, and environmental conditions lead to changes in system behavior [[6], [7], [8]]. Therefore, when designing the controller, their effect should be considered, and stability analysis should be performed in their presence [[9], [10], [11]].

In recent years, due to advances in communication science in the field of online storage of input and output data, several data-driven control methods have been proposed for nonlinear and complex systems [[12], [13], [14]]. In these methods, the basis of control signal design depends only on input and output data. For example, the methods of random approximation of simultaneous deviation [[15], [16], [17]] correlation-based adjustment [[18], [19], [20]] and iterative adjustment and feedback [[21], [22], [23]] are examples of data-driven control methods [[24], [25], [26]]. Due to the very useful features of data-driven controllers, including the lack of dependence on the system model, several effective data-driven methods for nonlinear systems in the presence of system constraints have been proposed [27]. For example, in Ref. [28], the iterative learning algorithm for the problem of tracking a nonlinear system involved with the saturation phenomenon is presented. Zhan et al. [29] proposed an adaptive dynamic programming algorithm to solve the operator saturation problem. In Ref. [30], a No model adaptive controller with an estimating structure for continuous time nonlinear systems is presented in which the input change rate limit is considered. One of the highlights of this study is the extension of No model adaptive control to continuous time systems.

Among all data-driven methods, the non-model adaptive method is recognized as a highly efficient method. This method is based on the concept of dynamic linearization. In this method, a dynamic linear estimate of the system is first provided by an online identification structure and then, according to the estimated model, the controller design is obtained based on several cost functions [[31], [32], [33]]. The presented dynamic estimation is divided into three forms: compact, partial, and complete. The difference between these forms is that the relationship between output and input at any given moment depends on the input and output of the previous moments [[34], [35], [36]]. For example, if only the output is dependent on the input of the previous moment, this state is called compressed form. Recently, due to the consistency of the adaptive control method without the model, this method has received much attention. For example, in Ref. [37] a comparative method without a compact model is presented for a nonlinear system with output saturation limit. Two No model adaptive algorithms for a specific class of single-input and single-output systems are presented in Ref. [38]. Also, in Refs. [[39], [40], [41]], a No model adaptive control method for the class of discrete-time nonlinear systems with quantization constraints and output saturation is presented.

Fuzzy control systems have gained significant attention with various applications in recent years [[42], [43], [44]]. An adaptive controller without a predictive model is proposed in Ref. [45] to solve the problem of nonlinear system stability in the presence of cyber-attacks. In Ref. [46], the problem of adaptive controller stability without a model during the disappearance of measurement signals is investigated and a relationship between the tracking error and the disappearance coefficient of the measurement signal is stated. In addition, in Refs. [[47], [48], [49]], an adaptive algorithm without a predictive model has been developed for discrete-time nonlinear systems, in none of which the issue of output saturation is considered. It should be noted that due to the ability of predictive control that is widely used in industry, adaptive control without predictive model can be a very useful option for unknown nonlinear systems with saturation limits [[50], [51], [52]]. According to the authors' knowledge, the subject of adaptive control design without predictive model in the presence of output saturation has not been worked on in the past literature [[53], [54], [55]].

In this paper, a predictive adaptive data axis controller for time-discrete nonlinear systems in the presence of output saturation constraints is proposed. First, considering that only input data and output saturated data are available, a new linear dynamic model based on the concept of partial derivative, it is presented and then according to the obtained model, an adaptive structure without a predictive model is presented. Since the proposed method uses saturated output data, the proposed method is more efficient than the usual adaptive methods without a predictive model. Also, due to the independence of the proposed method from the system model, the proposed method is more robust against model uncertainties than model-based methods. At first glance, the proposed method may be very close to the adaptive control method without a predictive model, but the presence of output saturation data leads to the definition of a new dynamic system and makes stability analysis much more difficult than the adaptive method without a predictive model. The main contributions of this work are summarized below.

1. The phenomenon of output saturation in intelligent and data-based control methods is not usually investigated, but it is investigated in detail herein.
2. To analyze the output saturation, a new dynamic linear model is introduced to behave like physical systems.
3. A type-3 fuzzy system with very high function approximation ability is utilized to estimate control system coefficients, allowing the tracking error to converge to zero faster.
4. The stability of the control system and guaranteeing its resilience in the face of parameter changes and output saturation is analyzed.

In the second part of the paper, the problem statement and the transfer model are described, and then the proposed predictive method is explained along with its stability analysis in the third part. Furthermore, in the fourth part, several simulations confirm the advantages of the proposed method over other methods.

## 2 Problem statement

Consider a nonlinear single-input and single-output system (1):

(1) $$y\left(p+1\right)=F\left(y\left(p\right),\ldots ,y\left(p-n_{y}\right),u\left(p\right),\ldots ,u\left(p-n_{u}\right)\right)$$

where F(0) represents the unknown nonlinear function and $n_{y}$ and $n_{u}$ represent the unknown degrees of input and output, respectively. Given that the proposed method is based on dynamic linearization, the following assumptions are expressed for use in trick 1 [[56], [57], [58]].

Assumption 1 For all instances the partial derivative of the function F(0) is relative to the input.

Assumption 2 Global Lipstick Company for system (1) in the sense that for all the moments that Δu(p)≠0 is:

|Δy(p+1)|≤L|Δu(p)|

So that Δu(p)=u(p)−u(p−1).

Δy(p+1)=y(p+1)−y(p),andL>0

Note 1 Both conditions above are logical and common conditions in adaptive control methods [11]. For example, the second hypothesis states that energy changes at the system output are a factor of energy changes at the system input. This assumption holds true for many real industrial processes, such as thermal and chemical processes.

Lemma 1 *If*|Δu(p)|≠0*and*Assumption 1, Assumption 2 hold for system (1), then there is a variable parameter with time Φ(p)∈R that can linearize system (1) to system (2).

(2) Δy(p+1)=Φ(p)Δu(p)

|Φ(p)|≤b

In system (2), b∈R. For more information, please refer to reference [10].

Note 2 With the help of trick 1, the nonlinear system (1) can be linearized in dynamic system (2). Now it is necessary to check the stability of trick 1 when the output saturation phenomenon occurs. For this purpose, with the help of the function Sat (0), the output saturation herein is defined as:

(3) $$z\left(p\right)=\text{Sat}\left(y\left(p\right)\right)=\left\{ \begin{array}{c} -Z_{0}\;y\left(p\right)<-Z_{0}\\ y\left(p\right)-Z_{0}\leq y\left(p\right)\leq Z_{0}\\ Z_{0}\;y\left(p\right)>Z_{0} \end{array}\right.$$

**Fig. 1:**
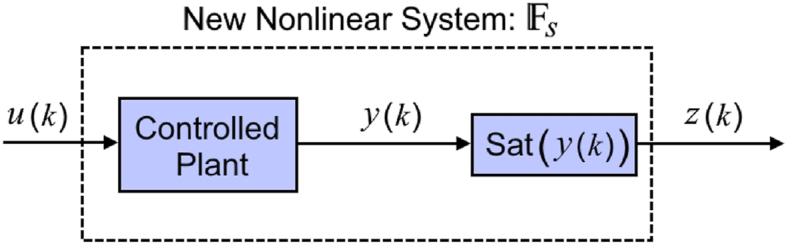
New structure of nonlinear system under control after considering output saturation.

In equation (3), z(p) and $Z_{0}$, express the output and the saturation level of the output, respectively. From Fig. 1, considering the effect of saturation, the output of system (1) becomes system (4):

(4) $$z\left(p+1\right)=F_{s}\left(z\left(p\right),\ldots ,z\left(p-n_{y}\right),u\left(p\right),\ldots ,u\left(p-n_{u}\right)\right)$$

In system (4), $Z\left(p\right)\in R^{1}$ and $F_{s}\left(0\right)$ is an unknown nonlinear function that has the nonlinear effect of output saturation.

Now, for system (4) to be transferred to the dynamic linear form, in trick 2 it is proved that trick 1 can also be used for system (4) and a new dynamic linear form can be presented for system (4) that the effect of output saturation in it is included.

Lemma 2 *If spaces* 1 *and* 2 *are established for system* (1) *and is rewritten to system* (4) *consideri*ng the saturation of the output of system (1). System (4) can then be rewritten in the form of dynamic linearization (5).

(5) $$\Delta z\left(p+1\right)=\Phi_{s}\left(p\right)\Delta u\left(p\right)$$

$$|\Phi_{s}\left(p\right)|\leq C$$

So that the fixed C∈R is limited.

Proof According to the definition of a partial derivative, the partial derivative of the function $F_{s}\left(z\left(p\right),u\left(p\right)\right)$ with respect to the input at any moment is equal to:

$$\frac{\partial F_{s}\left(z\left(p\right),u\left(p\right)\right)\;}{\partial u\left(p\right)}{|}_{z\left(p\right)}=cte$$

Therefore, as it is clear in the above definition, when deriving from one variable in multivariate functions, other variables are considered as constants. Here, when deriving from the input, the output variable is considered constant, so the occurrence of the output saturation phenomenon has no effect on the derivability of the function relative to the input and only affects the value of the derivative. Therefore, if the partial derivative of system (1) is present relative to the input, then system (4) is also derivable relative to the input. On the other hand,

|Δz(p)|≤|Δy(p)|

Therefore, according to Hypothesis 2, there is a C that satisfies the following conditions.

|Δz(p)|≤C|Δu(p)|

Therefore, if the universal Lip Sheet condition is met for system 1, then this condition is also met for system (4), and system (4) can be written in system (5).

## 3 Type-3 fuzzy system

To enhance the accuracy, Type-3 Fuzzy System is used as an estimator. The equations of the Type-3 Fuzzy System are as below.

1) Error and its derivative are assumed as inputs, $\mu_{1}=e$ and $\mu_{2}=\dot{e}$.
2) The membership ${\bar{P}}_{{\tilde{\Psi }}_{i|{\bar{z}}_{k}}^{j}}$, ${\bar{P}}_{{\tilde{\psi }}_{i|{\underline{z}}_{k}}^{j}}$, $P_{{\tilde{\Psi }}_{i|{\bar{n}}_{k}}^{j}}$ and $P_{{\tilde{\Psi }}_{i|m_{k}}^{j}}$ for ${\tilde{\Psi }}_{i}^{j}$ (j-th membership function (MF) for $\mu_{i},i=1,2$) are obtained:

(6) $$\begin{array}{c} {\bar{P}}_{{\tilde{\Psi }}_{i|{\bar{z}}_{k}}^{j}}= \end{array}\left\{ \begin{array}{c} 1-{\left(\frac{\left|\mu_{i}-b_{{\tilde{\Psi }}_{i}^{j}}\right|}{{\underline{f}}_{{\tilde{\Psi }}_{i}^{j}}}\right)}^{{\bar{p}}_{k}}\;\text{if}\;b_{{\tilde{\Psi }}_{i}^{j}}-{\underline{f}}_{{\tilde{\Psi }}_{i}^{j}}<\mu_{i}\leq b_{{\tilde{\Psi }}_{i}^{j}}\\ 1-{\left(\frac{\left|\mu_{i}-b_{{\tilde{\Psi }}_{i}^{j}}\right|}{{\bar{f}}_{{\tilde{\Psi }}_{i}^{j}}}\right)}^{{\bar{p}}_{k}}\;\text{if}\;b_{{\tilde{\Psi }}_{i}^{j}}<\mu_{i}\leq b_{{\tilde{\Psi }}_{i}^{j}}+{\bar{f}}_{{\tilde{\Psi }}_{i}^{j}}\\ 0\;\text{if}\;\mu_{i}>b_{{\tilde{\Psi }}_{i}^{j}}+{\bar{f}}_{{\tilde{\Psi }}_{i}^{j}}\;\text{or}\;\mu_{i}\leq b_{{\tilde{\Psi }}_{i}^{j}}-{\underline{f}}_{{\tilde{\Psi }}_{i}^{j}} \end{array}\right.$$

(7) $${\bar{P}}_{{\tilde{\psi }}_{i|{\underline{z}}_{k}}^{j}}=\left\{ \begin{array}{c} 1-{\left(\frac{\left|\mu_{i}-b_{{\tilde{\Psi }}_{i}^{j}}\right|}{{\underline{f}}_{{\tilde{\Psi }}_{i}^{j}}}\right)}^{p_{k}}\;\text{if}\;b_{{\tilde{\Psi }}_{i}^{j}}-{\underline{f}}_{{\tilde{\Psi }}_{i}^{j}}<\mu_{i}\leq b_{{\tilde{\Psi }}_{i}^{j}}\\ 1-{\left(\frac{\left|\mu_{i}-b_{{\tilde{\Psi }}_{i}^{j}}\right|}{{\bar{f}}_{{\tilde{\Psi }}_{i}^{j}}}\right)}^{{\underline{p}}_{k}}\;\text{if}\;b_{{\tilde{\Psi }}_{i}^{j}}<\mu_{i}\leq b_{{\tilde{\Psi }}_{i}^{j}}+{\bar{f}}_{{\tilde{\Psi }}_{i}^{j}}\\ \;\text{if}\;\mu_{i}>b_{{\tilde{\Psi }}_{i}^{j}}+{\bar{f}}_{{\tilde{\Psi }}_{i}^{j}}\;\text{or}\;\mu_{i}\leq b_{{\tilde{\Psi }}_{i}^{j}}-{\underline{f}}_{{\tilde{\Psi }}_{i}^{j}} \end{array}\right.$$

(8) $$P_{{\tilde{\Psi }}_{i|{\bar{n}}_{k}}^{j}}=\left\{ \begin{array}{c} 1-{\left(\frac{\left|\mu_{i}-b_{{\tilde{\Psi }}_{i}^{j}}\right|}{{\underline{f}}_{{\tilde{\Psi }}_{i}^{j}}}\right)}^{\frac{1}{p_{k}}}\;\text{if}\;b_{{\tilde{\Psi }}_{i}^{j}}-{\underline{f}}_{{\tilde{\Psi }}_{i}^{j}}<\mu_{i}\leq b_{{\tilde{\Psi }}_{i}^{j}}\\ 1-{\left(\frac{\left|\mu_{i}-b_{{\tilde{\Psi }}_{i}^{j}}\right|}{{\bar{f}}_{{\tilde{\Psi }}_{i}^{j}}}\right)}^{\frac{1}{p_{k}}}\;\text{if}\;b_{{\tilde{\Psi }}_{i}^{j}}<\mu_{i}\leq b_{{\tilde{\Psi }}_{i}^{j}}+{\bar{f}}_{{\tilde{\Psi }}_{i}^{j}}\\ \;\text{if}\;\mu_{i}>b_{{\tilde{\Psi }}_{i}^{j}}+{\bar{f}}_{{\tilde{\Psi }}_{i}^{j}}\;\text{or}\;\mu_{i}\leq b_{{\tilde{\Psi }}_{i}^{j}}-{\underline{f}}_{{\tilde{\Psi }}_{i}^{j}} \end{array}\right.$$

(9) $$P_{{\tilde{\Psi }}_{i|m_{k}}^{j}}=\left\{ \begin{array}{c} 1-{\left(\frac{\left|\mu_{i}-b_{{\tilde{\Psi }}_{i}^{j}}\right|}{{\underline{f}}_{{\tilde{\Psi }}_{i}^{j}}}\right)}^{\frac{1}{{\underline{p}}_{k}}}\;\text{if}\;b_{{\tilde{\Psi }}_{i}^{j}}-{\underline{f}}_{{\tilde{\Psi }}_{i}^{j}}<\mu_{i}\leq b_{{\tilde{\Psi }}_{i}^{j}}\\ 1-{\left(\frac{\left|\mu_{i}-b_{{\tilde{\Psi }}_{i}^{j}}\right|}{{\bar{f}}_{{\tilde{\Psi }}_{i}^{j}}}\right)}^{\frac{1}{p_{k}}}\;\text{if}\;b_{{\tilde{\Psi }}_{i}^{j}}<\mu_{i}\leq b_{{\tilde{\Psi }}_{i}^{j}}+{\bar{f}}_{{\tilde{\Psi }}_{i}^{j}}\\ \;\text{if}\;\mu_{i}>b_{{\tilde{\Psi }}_{i}^{j}}+{\bar{f}}_{{\tilde{\Psi }}_{i}^{j}}\;\text{or}\;\mu_{i}\leq b_{{\tilde{\Psi }}_{i}^{j}}-{\underline{f}}_{{\tilde{\Psi }}_{i}^{j}} \end{array}\right.$$

In equations (6), (7), (8), (9), ${\bar{p}}_{k}$/ ${\underline{p}}_{k}$ denotes upper/lower horizontal slice.

3) The l-th rule firings ${\bar{\Omega }}_{{\bar{p}}_{k}}^{l}$, ${\bar{\Omega }}_{{\underline{p}}_{k}}^{l}$, ${\underline{\Omega }}_{{\bar{p}}_{k}}^{l}$ and ${\underline{\Omega }}_{{\underline{p}}_{k}}^{l}$ are obtained as equations (10), (11), (12), (13).

(10) $${\bar{\Omega }}_{{\bar{p}}_{k}}^{l}={\bar{P}}_{{\tilde{\Psi }}_{1|{\bar{p}}_{k}}^{q_{1}}}\cdot {\bar{P}}_{{\tilde{\Psi }}_{1|{\bar{p}}_{k}}^{q_{2}}}\cdots {\bar{P}}_{{\tilde{\Psi }}_{1|{\bar{p}}_{k}}^{q_{n}}}$$

(11) $${\bar{\Omega }}_{{\underline{p}}_{k}}^{l}={\bar{P}}_{{\tilde{\Psi }}_{1|\underline{p}k}^{q_{1}}}\cdot {\bar{P}}_{{\tilde{\Psi }}_{1|{\underline{p}}_{k}}^{q_{2}}}\cdots {\bar{P}}_{{\tilde{\Psi }}_{1|{\underline{p}}_{k}}^{q_{n}}}$$

(12) $${\underline{\Omega }}_{{\bar{p}}_{k}}^{l}={\underline{P}}_{{\tilde{\Psi }}_{1|{\bar{p}}_{k}}^{q_{1}}}\cdot {\underline{P}}_{{\tilde{\Psi }}_{1|{\bar{p}}_{k}}^{q_{2}}}\cdots {\underline{P}}_{{\tilde{\Psi }}_{1|{\bar{p}}_{k}}^{q_{n}}}$$

(13) $${\underline{\Omega }}_{{\underline{p}}_{k}}^{l}={\underline{P}}_{{\tilde{\Psi }}_{1|\underline{p}k}^{q_{1}}}\cdot {\underline{P}}_{{\tilde{\Psi }}_{1}^{q_{2}}{\underline{p}}_{k}}\cdots {\underline{P}}_{{\tilde{\Psi }}_{1}^{q_{n}}|\underline{p_{k}}}$$

4) The output of Type-3 fuzzy system is given by equation (14).

(14) $$T3-FLS=\sum_{k=1}^{K}\;\left({\underline{p}}_{k}{\underline{S}}_{k}+{\bar{p}}_{k}{\bar{S}}_{k}\right)/\sum_{k=1}^{K}\;\left({\underline{p}}_{k}+{\bar{p}}_{k}\right)$$

where ${\bar{S}}_{k}$ and ${\underline{S}}_{k}$ are given respectively by equations (15), (16).

(15) $${\bar{S}}_{k}=\frac{\sum_{l=1}^{P}\;\left({\bar{\Omega }}_{{\bar{p}}_{k}}^{l}{\bar{w}}_{l}+{\underline{\Omega }}_{{\bar{p}}_{k}}^{l}{\underline{w}}_{l}\right)}{\sum_{l=1}^{P}\;\left({\bar{\Omega }}_{{\bar{p}}_{k}}^{l}+\Omega_{{\bar{p}}_{k}}^{l}\right)}$$

(16) $${\underline{S}}_{k}=\frac{\sum_{l=1}^{P}\;\left({\bar{\Omega }}_{{\underline{p}}_{k}}^{l}{\bar{w}}_{l}+{\underline{\Omega }}_{{\underline{p}}_{k}}^{l}{\underline{w}}_{l}\right)}{\sum_{l=1}^{P}\;\left({\bar{\Omega }}_{p_{k}}^{l}+{\underline{\Omega }}_{p_{k}}^{l}\right)}$$

The presented rules are regulated by equation (17).

(17) $$\begin{array}{c} {\bar{w}}_{l}\left(t+1\right)={\bar{w}}_{l}\left(t\right)+\frac{1}{\sum_{k=1}^{K}\;\left({\underline{p}}_{k}+{\bar{p}}_{k}\right)}\sum_{k=1}^{K}\;\frac{{\bar{p}}_{k}{\bar{\Omega }}_{{\bar{p}}_{k}}^{l}}{\sum_{l=1}^{P}\;\left({\bar{\Omega }}_{{\bar{p}}_{k}}^{l}+{\underline{\Omega }}_{{\bar{p}}_{k}}^{l}\right)}\;+\frac{1}{\sum_{k=1}^{K}\;\left({\underline{p}}_{k}+{\bar{p}}_{k}\right)}\sum_{k=1}^{K}\;\frac{{\underline{p}}_{k}{\bar{\Omega }}_{{\underline{p}}_{k}}^{l}}{\sum_{l=1}^{P}\;\left({\bar{\Omega }}_{{\underline{p}}_{k}}^{l}+{\underline{\Omega }}_{{\underline{p}}_{k}}^{l}\right)}\\ {\underline{w}}_{l}\left(t+1\right)={\underline{w}}_{l}\left(t\right)+\frac{1}{\sum_{k=1}^{K}\;\left({\underline{p}}_{k}+{\bar{p}}_{k}\right)}\sum_{k=1}^{K}\;\frac{{\bar{p}}_{k}{\underline{\Omega }}_{{\bar{p}}_{k}}^{l}}{\sum_{l=1}^{P}\;\left({\bar{\Omega }}_{{\bar{p}}_{k}}^{l}+{\underline{\Omega }}_{{\bar{p}}_{k}}^{l}\right)}\;+\frac{1}{\sum_{k=1}^{K}\;\left({\underline{p}}_{k}+{\bar{p}}_{k}\right)}\sum_{k=1}^{K}\;\frac{{\underline{m}}_{k}{\underline{\Omega }}_{{\underline{m}}_{k}}^{l}}{\sum_{l=1}^{M}\;\left({\bar{\Omega }}_{{\underline{p}}_{k}}^{l}+{\underline{\Omega }}_{{\underline{p}}_{k}}^{l}\right)} \end{array}$$

## 4 Data-based control method

This section presents a predictive adaptive data-driven controller for system (1) in the presence of output saturation. According to equation (5), the output of the next few steps can be defined as (18).

(18) $$\left\{ \begin{array}{c} z\left(p+1\right)=z\left(p\right)+\Phi_{s}\left(p\right)\Delta u\left(p\right)\\ z\left(p+2\right)=z\left(p+1\right)+\Phi_{s}\left(p+1\right)\Delta u\left(p+1\right)\\ .\\ .\\ .\\ z\left(p+N\right)=z\left(p+N-1\right)\Phi_{s}\left(p+N-1\right)\Delta u\left(p+N-1\right)\\ =z\left(p+N-2\right)+\Phi_{s}\left(p+N-2\right)\Delta u\left(p+N-2\right)+\Phi_{s}\left(p+N-1\right)\Delta u\left(p+N-1\right) \end{array}\right.$$

Mode equation (18) can be rewritten as equation (19).

(19) $$Z_{N}\left(p+1\right)=E\left(p\right)z\left(p\right)+A_{1}\left(p\right)\Delta U_{Nu}\left(p\right)$$

So that:

(20) $$A_{1}\left(k\right)={\left[\begin{array}{cccc} \Phi_{s}\left(p\right) & 0 & 0 & 0\\ \Phi_{s}\left(p\right) & \Phi_{s}\left(p+1\right) & 0 & 0\\ \vdots & \vdots & \ddots & \vdots \\ \Phi_{s}\left(p\right) & \Phi_{s}\left(p+1\right) & \ddots & \Phi_{s}\left(p+N_{u}-1\right)\\ \vdots & \vdots & \cdots & \vdots \\ \Phi_{s}\left(p\right) & \Phi_{s}\left(p+1\right) & \ldots & \Phi_{s}\left(p+N_{u}-1\right) \end{array}\right]}_{N\times N_{u\;}}$$

(21) $$E\left(p\right)={\left[1,1,\cdots ,1\right]}_{1\times N}^{T}$$

(22) $$R_{N}\left(p+1\right)={\left[R\left(p+1\right),\ldots ,R\left(p+N\right)\right]}^{T}$$

also $Z_{N}\left(p+N\right)$, $R_{N}\left(p+1\right)$, and $\Delta U_{N_{u}}\left(p\right)={\left[\Delta u\left(p\right),\ldots ,\Delta u\left(p+N_{u}-1\right)\right]}^{T}$. The predicted output vector is the reference signal vector on the control horizon and the control vector, respectively. In addition, in relation (19), N and $N_{u}$ are the forecast horizon and the control horizon. Now, to obtain the appropriate control signal, the cost function can be considered in equation (23).

(23) $$J={\left[R_{N}\left(p+1\right)-Z_{N}\left(p+1\right)\right]}^{T}\left[R_{N}\left(p+1\right)-Z_{N}\left(p+1\right)\right]+\lambda \Delta U_{N_{u}}^{T}\Delta U_{N_{u}}\left(p\right)$$

Due to the high-cost function, the control signal is obtained in the following form (see Ref. [21]).

$$\Delta U_{N_{u}}\left(p\right)={\left[A_{1}^{T}A_{1}\left(p\right)+\lambda I\right]}^{-1}\times A_{1}^{T}\left(p\right)\left[R_{N}\left(p+1\right)-E\left(p\right)z\left(p\right)\right]$$

In this regard, λ is the penalty coefficient and plays an important role in the stability of the controlled system in the presence of saturated output. Next, considering the law of forecast horizon, the control signal at moment p is obtained as follows.

(24) $$u\left(p\right)=u\left(p-1\right)+G^{T}\Delta U_{N_{u}}\left(p\right)$$

In equation (24), $G^{T}=\left[1,0,\ldots ,0\right]$.

As can be seen in equation (20), matrix $A_{1}\left(p\right)$ contains elements $\Phi_{s}\left(p\right),\Phi_{s}\left(p+1\right),\ldots ,\Phi_{s}\left(p+N_{u}‐1\right)$ that must be estimated. In this paper, $\Phi_{s}\left(k\right)$, is estimated through equation (25) [21].

(25) $${\hat{\Phi }}_{s}\left(p\right)={\hat{\Phi }}_{s}\left(p-1\right)+\frac{\eta \Delta u\left(p-1\right)}{\mu +\Delta u{\left(p-1\right)}^{2}}\left(\Delta z\left(p\right)-{\hat{\Phi }}_{s}\left(p-1\right)\Delta u\left(p-1\right)\right)$$

So that μ>0 and 0<η≤1 are the penalty coefficient and the step coefficient, respectively. The estimation algorithm should also be used to estimate the other elements of the $A_{1}\left(k\right)$ matrix according to the k moment information.

Note 3 In Ref. [21], a multi-level hierarchical forecasting algorithm is proposed for estimation. Therefore, in the proposed structure, equation (26) is used to estimate the elements of the $A_{1}\left(p\right)$ matrix.

(26) $${\hat{\Phi }}_{s}\left(p+j\right)=\psi_{1}\left(p\right){\hat{\Phi }}_{s}\left(p+j‐1\right)+\psi_{2}\left(p\right){\hat{\Phi }}_{s}\left(p+j‐2\right)+\cdots +\psi_{n_{i}}\left(p\right){\hat{\Phi }}_{s}\left(p+j‐n_{i}\right)$$

where $n_{p}$ is a constant degree, which is normally considered to be between 2 and 7.

Note 4 As can be seen in equations (18), (19), (20), (21), (22), the proposed structure is at first glance very similar to the current structure of adaptive control without a predictive model. But the presence of output saturation signal in the relationships causes a difference, which makes the stability analysis more difficult. In general, the structure of the proposed method is given together by equations (27), (28), (29), (30), (31), (32).

(27) $${\hat{\Phi }}_{s}\left(p\right)={\hat{\Phi }}_{s}\left(p-1\right)+\frac{\eta \Delta u\left(p-1\right)}{\mu +\Delta u{\left(p-1\right)}^{2}}\left(\Delta z\left(p\right)-{\hat{\Phi }}_{s}\left(p-1\right)\Delta u\left(p-1\right)\right)$$

(28) $${\hat{\Phi }}_{s}\left(p\right)={\hat{\Phi }}_{s}\left(1\right)\;\text{if}\;{\hat{\Phi }}_{s}\left(p\right)\leq e\;\text{or}\;\text{sign}\left({\hat{\Phi }}_{s}\left(p\right)\right)\neq \text{sign}\left({\hat{\Phi }}_{s}\left(1\right)\right)$$

(29) $$\psi \left(p\right)=\psi \left(p‐1\right)+\frac{\hat{j}\left(p‐1\right)}{\delta +\hat{j}{\left(p‐1\right)}^{T}}\left[{\hat{\Phi }}_{s}\left(p\right)‐{\hat{Ϛ}}^{T}\left(p‐1\right)\psi \left(p‐1\right)\right]$$

(30) ψ(p)=ψ(1)ifψ(p)≤M

(31) $${\hat{\psi }}_{s}\left(p+j\right)=\psi_{1}\left(p\right){\hat{\Phi }}_{s}\left(p+j‐1\right)+\psi_{2}\left(p\right){\hat{\Phi }}_{s}\left(p+j‐2\right)+\ldots +\psi_{n_{i}}\left(p\right){\hat{\Phi }}_{s}\left(p+j‐n_{i}\right)$$

(32) $$\Delta U_{N_{u}}\left(p\right)={\left[A_{1}^{T}A_{1}+\lambda I\right]}^{-1}A_{1}^{T}\left(p\right)\left[R_{N}\left(p+1\right)-E\left(p\right)z\left(p\right)\right]$$

where $\Delta z\left(p\right)=z\left(p\right)-z\left(p-1\right),\hat{j}\left(p-1\right)={\left[{\hat{\Phi }}_{s}\left(p-1\right),\cdots ,{\hat{\Phi }}_{s}\left(p-n_{i}\right)\right]}^{T},\eta \epsilon (0,1],\delta \epsilon (0\text{,}1],\varepsilon >0,M>0,\lambda >0$. In addition, ε and M are small constants used in the reset mechanism. To prove the stability of the proposed method, the output error and follow-up error variables are defined in equations (33), (34), respectively [59,60].

(33) ε(p)≜R(p)−Z(p)

(34) e(p)≜R(p)−y(p)

According to relation (3), the relationship between output and follow-up errors can be written in equation (35) [61].

(35) ε(p)=γ(p)e(p),0<γ(p)≤1

where ε(p) is output error at the sampling point at point p, e(p) is the follow-up error at the sampling point p and γ(p) is a proportionality factor that scales the follow-up error e(p) to the output error ε(p).

Lemma 3 *Acc*ording to the definition of the saturation function (3), the relationship between sensor changes and actual output changes can be considered in equation (36).

(36) Δz(p)=g(p)Δy(p),0≤g(p)≤1

The proof of this trick is stated in Ref. [10].

Lemma 4 In [21], it is proven that there exists an $\lambda_{\min}>0$ that holds for the following inequalities for $\lambda >\lambda_{\min}$ and 0<B≤1.

$$0<|1-\Phi \left(p\right)G^{T}{\left[A_{1}^{T}A_{1}\left(p\right)+\lambda I\right]}^{-1}\;A_{1}^{T}\left(p\right)E\left(p\right)|\leq B<1$$

Now, with the help of defined variables and 3–4 slides, the stability of the proposed control structure in Theory 1 can be proven. Also, since the main purpose of this paper is to design a predictive adaptive data-driven controller for a nonlinear system in the presence of output saturation, the following assumption is considered on the reference signal before stating the theory.

Assumption 3 It is assumed that the arbitrary reference signal R(p) is in the range between saturation levels. That means $|R\left(p\right)|\leq Z_{0}$.

Theorem 1 *If*Assumption 1, Assumption 2, Assumption 3*are established* for system (4) and the proposed control structures (15) and (20) are applied to the system, then there exists an $0<\lambda_{\min}$ that holds the following equations for $\lambda >\lambda_{\min}$.

1. *For fixed reference signal*:

$$\lim_{k\to \infty }e\left(p\right)=0$$

2. For time-varying signals and condition $\Delta R\left(p\right)<b_{3}$,

$$\lim_{k\to \infty }e\left(p\right)=b_{3}$$

where ΔR(p) represents the changes in the reference signal.

Note 5 Before starting to prove Theory 1, it is assumed that for Δu(p)≠0 the sign $\Phi_{s}\left(p\right)$ always remains constant. This means that it is a positive value for all moments $\Phi_{s}\left(p\right)>\bar{\theta }>0$ and $\bar{\theta }$. This assumption is a very common assumption in no model adaptive controllers [11].

**Proof**: First, the convergence of the ${\hat{\Phi }}_{s}\left(p\right)$ estimation algorithm for the following two cases is proved.

The first case:

(37) $${\hat{\Phi }}_{s}\left(p\right)\leq e\;\text{or}\;\text{sign}\left({\hat{\Phi }}_{s}\left(p\right)\right)\neq \text{sign}\left({\hat{\Phi }}_{s}\left(1\right)\right)$$

**The second case:**$e\leq {\hat{\Phi }}_{s}\left(p\right)$.

**The first case**: In this mechanism they reset and take the estimated value to a predetermined value, so in this case the limitation of ${\hat{\Phi }}_{s}\left(p\right)$ is obvious.

**The second case:** In this case, the parameter estimation error is defined as follows.

$${\tilde{\Phi }}_{s}\left(p\right)={\hat{\Phi }}_{s}\left(p\right)-\Phi_{s}\left(p\right)$$

Equation (27) can be rewritten as

(38) $${\tilde{\Phi }}_{s}\left(p\right)=\left(1-\frac{\eta \Delta u^{2}\left(p-1\right)}{\mu +\Delta u^{2}\left(p-1\right)}\right){\tilde{\Phi }}_{s}\left(p-1\right)+\Delta \Phi_{s}\left(p\right)$$

Taking absolute values equation (38), results in relation (39) [62,63].

(39) $$\left|{\tilde{\Phi }}_{s}\left(p\right)\right|\leq \left|1-\frac{\eta \Delta u^{2}\left(p-1\right)}{\mu +\Delta u^{2}\left(p-1\right)}\right|\left|{\tilde{\Phi }}_{s}\left(p-1\right)\right|+\left|\Delta \Phi_{s}\left(p\right)\right|$$

It was also given that μ>0 and η∈(0,1), the relations (40) and then (41) hold.

(40) $$\eta \Delta u^{2}\left(p-1\right)<\Delta u^{2}\left(p-1\right)<\mu +\Delta u^{2}\left(p-1\right)$$

(41) $$0<1-\frac{\eta \Delta u^{2}\left(p-1\right)}{\mu +|\Delta u^{2}\left(p-1\right){|}^{2}}\leq C_{1}<1$$

On the other hand, $\left|\Phi_{s}\left(p\right)\right|\leq C$ is therefore [64]:

(42) $$\left|{\tilde{\Phi }}_{s}\left(p\right)\right|\leq C_{1}\left|{\tilde{\Phi }}_{s}\left(p-1\right)\right|+C+2C\leq C_{1}^{2}\left|{\tilde{\Phi }}_{s}\left(p-2\right)\right|+3C_{1}C+3C\leq C_{1}^{p-1}\left|{\tilde{\Phi }}_{s}\left(1\right)\right|+\frac{3C}{1-C_{1}}$$

Given that $\left|C_{1}\right|<1$, ${\tilde{\Phi }}_{s}\left(p\right)$ are constrained and the convergence of F is proved using relations (42) and (37). In the following, the convergence of the tracking error in the two modes of fixed reference signal and time-varying reference signal is proved.

### 4.1 Fixed reference signal

According to $R_{d}\left(p\right)=R_{d}\left(p+1\right)\;and\;E\left(p\right)={\left[1,1,\cdots ,1\right]}_{1\times N}^{T}$, equation (43) is obtained.

(43) $$R_{N}\left(p+1\right)-E\left(p\right)z\left(p\right)=E\left(p\right)\left(R_{d}\left(p+1\right)-z\left(p\right)\right)$$

Using (21), (31) and (20) can be written in equation (44) [65].

(44) $$\Delta U_{N_{u}}\left(p\right)={\left[A_{1}^{T}A_{1}\left(p\right)+\lambda I\right]}^{-1}A_{1}^{T}\left(p\right)E\left(p\right)\varepsilon \left(p\right)$$

According to the control signal, equation (45) holds.

(45) $$\left|\varepsilon \left(p+1\right)\right|=\left|R\left(p+1\right)-z\left(p+1\right)\right|=\left|R\left(p+1\right)-z\left(p\right)-\Phi_{s}\left(p\right)\Delta U_{N_{u}}\left(p\right)\right|$$

$$=\left|\varepsilon \left(p\right)-\Phi_{s}\left(p\right)G^{T}{\left[A_{1}^{T}A_{1}\left(p\right)+\lambda I\right]}^{-1}A_{1}^{T}\left(p\right)E\left(p\right)\varepsilon \left(p\right)\right|$$

Therefore, according to trick 4, there is a constant value of $C_{2}$ that establishes inequality (46).

(46) $$0<\left|1-\Phi_{s}\left(p\right)G^{T}{\left[A_{1}^{T}A_{1}\left(p\right)+\lambda I\right]}^{-1}A_{1}^{T}\left(p\right)E\left(p\right)\right|<C_{2}\leq 1$$

On the other hand,

(47) $$\left|\varepsilon \left(p+1\right)\right|\leq \left|1-\Phi_{s}\left(p\right)G^{T}{\left[A_{1}^{T}A_{1}\left(p\right)+\lambda I\right]}^{-1}A_{1}^{T}\left(p\right)E\left(p\right)\right|\left|\varepsilon \left(p\right)\right|$$

From relation (47) and according to (34) and , relation (48) holds.

(48) $$\left|\varepsilon \left(p+1\right)\right|\leq C_{2}\left|\varepsilon \left(p\right)\right|\leq \ldots \leq C_{2}^{p}\left|\varepsilon \left(1\right)\right|$$

This means $\lim_{p\to \infty }\varepsilon \left(p+1\right)=0$. Also, ε(p+1)=γ(p+1)e(p+1) and 0<γ(p+1)≤1, therefore,

$$\lim_{p\to \infty }e\left(p+1\right)=0$$

### 4.2 Time-varying reference signal

Assuming that $\Delta R_{d}\left(p\right)<C_{3}$, the follow-up error can be written as equation (49).

$$\left|\varepsilon \left(p+1\right)\right|=\left|R_{d}\left(p+1\right)-z\left(p\right)-\Phi_{s}\left(p\right)\Delta u\left(p\right)\right|$$

(49) $$\left|\varepsilon \left(p+1\right)\right|=\left|R_{d}\left(p\right)-z\left(p\right)-\Phi_{s}\left(p\right)\Delta u\left(p\right)+\Delta R_{d}\left(p\right)\right|$$

Therefore, relation (50) holds [66].

(50) $$\left|\varepsilon \left(p+1\right)\right|\leq C_{2}^{p}\left|\varepsilon \left(1\right)\right|+\left|\Delta R_{d}\left(p\right)\right|$$

So given that ε(p+1)=γ(p+1)e(p+1), relation (51) satisfies.

(51) $$\left|e\left(p+1\right)\right|\leq C_{2}^{p}\left|e\left(1\right)\right|+C_{3}$$

Thus, the pursuit error is entirely dependent on ${\;C}_{3}$.

Note 6 As can be seen in the proof, if the reference signal is time-varying and changes quite abruptly, the tracking error also increases abruptly. Also, if the reference signal is constant, the tracking error will decrease to zero over time. Note that if γ(k)=1, equation (52) holds:

(52) $$e\left(p+1\right)=\left|1-\Phi_{s}\left(p\right)G^{T}{\left[A_{1}^{T}A_{1}\left(p\right)+\lambda I\right]}^{-1}A_{1}^{T}\left(p\right)E\left(p\right)\right|\left|e\left(p\right)\right|$$

And if γ(p)<1, the following inequality holds.

$$\left|1-\Phi_{s}\left(p\right)G^{T}{\left[A_{1}^{T}A_{1}\left(p\right)+\lambda I\right]}^{-1}A_{1}^{T}\left(p\right)E\left(p\right)\right|<$$

(53) $$\left|1-\Phi_{s}\left(p\right)G^{T}{\left[A_{1}^{T}A_{1}\left(p\right)+\lambda I\right]}^{-1}A_{1}^{T}\left(p\right)E\left(p\right)\gamma \left(p\right)\right|$$

**Fig. 2:**
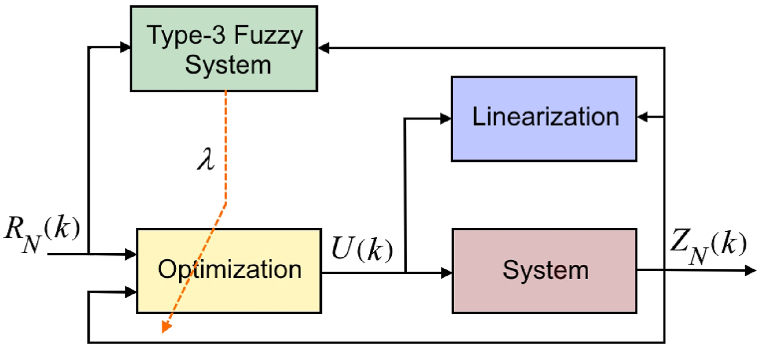
Proposed control System Diagram.

**Fig. 3:**
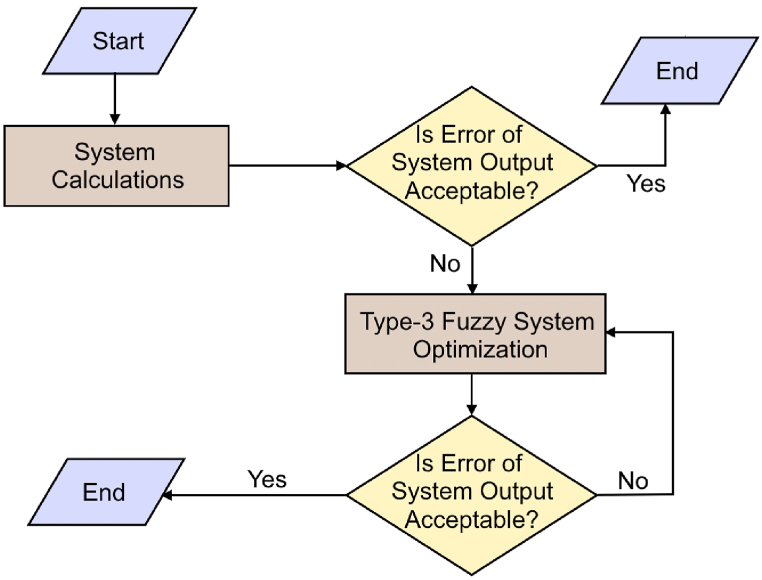
Simple flowchart of the proposed algorithm.

Inequality (53) indicates that the closer the saturation level is to the reference signal level, the slower the convergence rate. Fig. 2 shows the block diagram of the proposed control system. It is clear from the figure that Type-3 Fuzzy System has an important role in optimization section. A simple flowchart of the proposed model is also illustrated in Fig. 3.

## 5 Simulation results

In this section, some simulations are presented to show the efficiency and superiority of the proposed method in controlling nonlinear systems in the presence of output saturation over several data-driven methods such as PID and CFDL-MFAC. Consider a time-varying discrete nonlinear system given by relation (42) [21,22]. As can be seen, relation (42) is a nonlinear system with many time changes. Based on (14), (15), (16), (17), (18), (19), (20), an adaptive controller without a predictive model is designed to follow the time-varying reference signal by considering the effective parameters as follows.

$$\eta =0.1,\epsilon =10^{-4},\delta =1,\lambda =1,\mu =0.4,N_{p}=5,N_{u}=3$$

$${\hat{\Phi }}_{s}\left(1\right)=10,{\hat{\Phi }}_{s}\left(2\right)=10,{\hat{\Phi }}_{s}\left(3\right)=10,{\hat{\Phi }}_{s}\left(4\right)=10$$

$$y\left(p+1\right)=\left\{ \begin{array}{c} \frac{2.5y\left(p\right)y\left(kp1\right)}{1+y{\left(p\right)}^{2}+y{\left(p-1\right)}^{2}}+0.7\;\sin\left(0.5\left(y\left(p\right)+y\left(p-1\right)\right)\right)\times \cos\left(0.5\left(y\left(p\right)+y\left(p-1\right)\right)\right)\;+1.2u\left(p\right)+1.4\left(p-1\right)\;1\leq p\leq 250\;\\ \frac{2.5y\left(p\right)y\left(p-1\right)}{1+y{\left(p\right)}^{2}+y{\left(p-1\right)}^{2}}+0.7\;\sin\left(0.5\left(y\left(p\right)+y\left(p-1\right)\right)\right)\times \cos\left(0.5\left(y\left(p\right)+y\left(p-1\right)\right)\right)\;+1.2u\left(p-1\right)+1.4u\left(kp2\right)\;250\leq p\leq 500\;\\ \frac{5y\left(p\right)y\left(p-1\right)}{1+y{\left(p\right)}^{2}+y{\left(p-1\right)}^{2}+y{\left(p-2\right)}^{2}+u\left(p\right)+1.1u\left(p-1\right)}\;500\leq p\leq 750\;\\ -0.1y\left(p\right)-0.2y\left(p-1\right)-0.3\left(p-2\right)+0.1u\left(p-2\right)+0.02u\left(p-3\right)+0.03u\left(p-4\right)\;750\leq p\; \end{array}\right.$$

To compare the proposed method with other methods for system (43), a PID controller is considered whose parameters are set based on reference [21].

$$u\left(p\right)=P_{i}\left[e\left(p\right)+\frac{1}{T_{I}}\sum_{j=0}^{p}e\left(j\right)+T_{D}\left(e\left(p\right)-e\left(p-1\right)\right)\right]$$

**Fig. 4:**
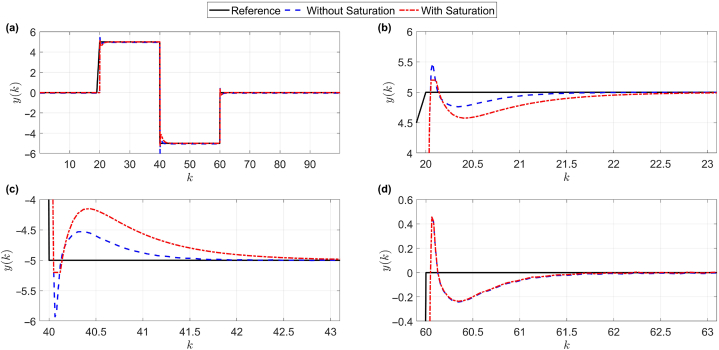
Performance of the proposed control system with and without output saturation. For more clarity, parts of panel (a) are enlarged in panels (b)–(d).

So that $T_{D}=1,T_{I}=1\;and\;P_{i}=0.1$ is [21]. The reference signal is also shown in Fig. 4, where panels (b), (c), and (d) are magnified sections of panel (a).

**Fig. 5:**
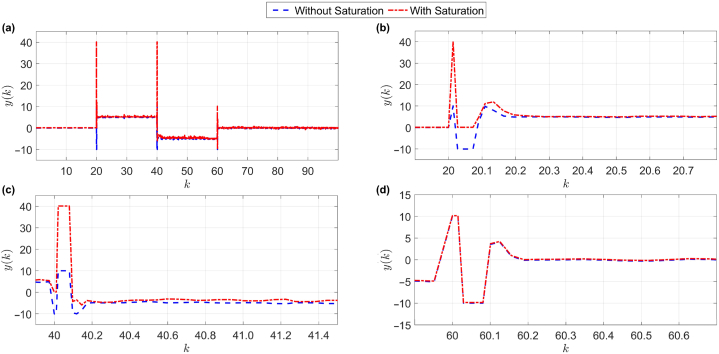
The control signal of the reference tracking shown in Fig. 4. For more clarity, parts of panel (a) are enlarged in panels (b)–(d).

Naturally, in any control system, the condition of the control signal is also important. Therefore, in Fig. 5, the control signal of the reference tracking shown in Fig. 4, is depicted. Again, panels (b), (c), and (d) are magnified sections of panel (a) in Fig. 5. Considering the response in Fig. 4, the control parameters in the following form are obtained:

$$\eta =0.1,\epsilon =10^{-4},\delta =1,\lambda =0.3,\mu =0.5,N_{P}=10,{\;N}_{u}=3\text{,}$$

$${\hat{\Phi }}_{s}\left(1\right)=29,{\hat{\Phi }}_{s}\left(2\right)=29,{\hat{\Phi }}_{s}\left(3\right)=29,{\hat{\Phi }}_{s}\left(4\right)=30$$

**Fig. 6:**
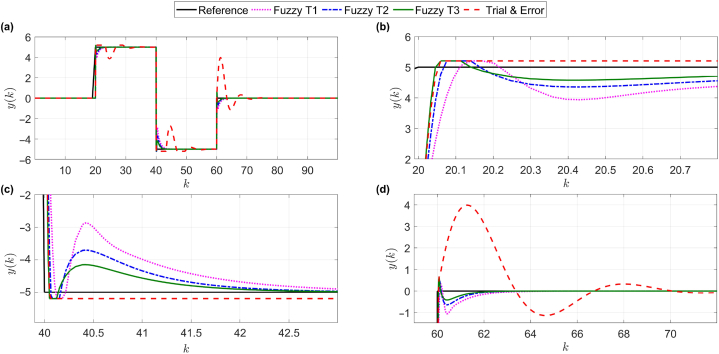
Comparison of the proposed control system (type-3 fuzzy) with other methods. For more clarity, parts of panel (a) are enlarged in panels (b)–(d).

To better evaluate the performance of the proposed method, a comparison with type-1 and type-2 fuzzy systems and control systems based on trial-and-error parameter tuning is made. In addition, in all methods, the phenomenon of output saturation is considered (see Fig. 6).

As shown in Fig. 6, the proposed controller, despite its nonlinear nature and time-varying system (42), was able to meet the main purpose of the paper (robust against saturation of the output and acceleration of the convergence error to zero) and it has a much better performance than the trial & error-based controller, see the zoomed panels (b), (c), and (d) of panel (a) in Fig. 6. Now, in addition to the main purpose of the article, another goal is to not exceed the output limits. As shown in Fig. 4, Fig. 5, Fig. 6, the answer obtained is slightly naked. The advantage of the proposed algorithm over the trial & error-based control method is that in the moments of saturation phenomenon, considering that the proposed method is adaptable, the dynamic controller detects the new situation and updates its dynamic model accordingly, then according to the obtained dynamic model, the control signal is generated in accordance with the predictive adaptive structure, which leads to an increase in the convergence speed of the pursuit research error relative to the trial & error-based control at the time of saturation.

Fig. 6 shows the importance of using computational intelligence in control systems. As can be seen, parameter estimation with fuzzy system has a significant effect on improving the efficiency of the control system. On the other hand, the increase in the accuracy of the fuzzy system is significant with the increase in its order, so type-3 fuzzy system (proposed method) provided the best suitable answer. To further compare the performance of the proposed method with other methods, in Table 1 two measures of root mean squares error (RMSE) and step response time are calculated.

**Table 1:** Comparison based on RMSE and step response time.

Method	RMSE	Step response time (s)
Trial & Error	2.427	11
Method of [32]	1.377	3.35
Type-1 Fuzzy	0.935	1.39
Method of [27]	0.792	1.11
Type-2 Fuzzy	0.758	1.16
Type-3 Fuzzy (Proposed method)	0.593	1.02

In general, the proposed controller, despite its nonlinear and time-varying nature, successfully achieves the main objectives outlined in the paper, notably robustness against output saturation and acceleration of convergence error to zero. It significantly outperforms the trial & error-based controller, particularly in adapting to saturation phenomena and updating its dynamic model accordingly for improved control signal generation. This highlights the importance of computational intelligence, particularly through parameter estimation with fuzzy systems, in enhancing control system efficiency. The type-3 fuzzy system, as part of the proposed method, offers the most suitable solution. Additionally, comparisons are made using measures, such as RMSE and step response time to further validate the superior performance of the proposed method.

## 6 Conclusions

In this paper, Type-3 fuzzy system-based model free control is presented for time-discrete nonlinear system. Required unknown parameters for control system are estimated by type-3 fuzzy system. The phenomenon of output saturation, which is a very important challenge in control systems, is considered in the presented method therefore a new linear dynamic model is introduced. Due to the use of saturated output data in the proposed method, its efficiency is higher than conventional methods. On the other hand, this method is more robust to model uncertainties than other methods. The simulation results fully confirmed the effectiveness of the proposed method. The value of RMSE and step response time of the proposed method is equal to 0.593 and 1.02s, respectively, which has a better result than other compared methods.

For future works, it is possible to develop type-3 fuzzy systems for other control methods, such as model predictive control, adaptive control, robust control, and optimal control. Utilizing evolutionary algorithms to optimally adjust the type-3 fuzzy system itself is another research gap. Moreover, researchers can find a solution to reduce type-3 fuzzy system calculations for use in online applications. For example, the structured learning model can be utilized to reduce the number of fuzzy rules and parameters. Utilizing this model, despite the decrease in accuracy, the number of calculations can be greatly reduced. The integration of machine learning algorithms, such as neural networks or reinforcement learning, may further enhance the adaptability and performance of the type-3 fuzzy system in real-time applications. Lastly, the use of fuzzy clustering for control of discrete-time nonlinear systems will also be explored in future studies [[67], [68], [69]].

## Funding

This research received no funding.

## Data and code availability

Data will be made available on request**.**

## CRediT authorship contribution statement

**Xuejun Zhou:** Writing – original draft, Methodology, Formal analysis, Conceptualization. **Ying Dai:** Writing – review & editing, Supervision, Conceptualization. **Ebrahim Ghaderpour:** Writing – review & editing, Visualization, Supervision, Conceptualization. **Ardashir Mohammadzadeh:** Writing – review & editing, Supervision, Conceptualization. **Pierpaolo D'Urso:** Writing – review & editing, Supervision, Conceptualization.

## Declaration of competing interest

The authors declare that they have no known competing financial interests or personal relationships that could have appeared to influence the work reported in this paper.
